# Reducing the Pressure on Mental Health Team by Improving Post-Discharge Follow-Up of Self-Harm or Suicidal Patients in Primary Care

**DOI:** 10.1192/bjo.2022.307

**Published:** 2022-06-20

**Authors:** Nafiz Imtiaz, John McLaughlin

**Affiliations:** Oakleaf Medical Practice, Londonderry, United Kingdom

## Abstract

**Aims:**

Northern Ireland has had the highest suicide and self-harm rate in the UK since 2012 according to National Statistics Office with 12.5 deaths per 100,000 population compared to 10.5 in the rest of the country. Evidence shows that the risk of suicide hugely increases following self-harm, and the greatest risk is immediately after the self-harm episode. Better access to health care, especially to primary care, in this period, can actively reduce the risk to this vulnerable patient group. Patients assessed for self-harm in the emergency department are often followed up by the mental health/crisis team. Due to lack of resources and staff shortages this is often not possible in a timely fashion. NICE suggests that patients should be offered a follow-up appointment in primary care within 48 hours of discharge. We aimed to ensure 70% of patients discharged from secondary care following an episode of suicidal ideation or self-harm are contacted proactively by mental health practitioner (MHP) or GP within 48 hours of communication from secondary care.

**Methods:**

The project underwent two PDSA cycles. An electronic workflow was created to provide easy patient identification, assessment and follow-up. A process mapping was done after discussion with the GPs, administrative team, practice nurses and MHP. Outcome was measured by finding out percentage of patients: 1) Contacted within 48 hours of communication following an episode of self-harm 2) Appropriately coded 3) Comprehensively assessed 4) Risk stratified and minimized following each cycle.

**Results:**

Over a period of three months, following two PDSA cycles, the frequency of these contacts increased from 0 to 80% (median) with an average 3.8 (83%) patients reviewed per week. The patient experience and satisfaction also improved significantly.

**Conclusion:**

General practice (GP) has long been known as the next of kin for patients in the health care system. As GP is mostly the first point of contact for the patients, it can contribute significantly to ease the rising pressure on the mental health team. Also, a small number of weekly contacts from each GP can make a huge difference in nationwide patient safety and experience. We hope this intervention will significantly improve patient safety and reduce further self-harm presentation to ED in the long run.

